# Osteogenic capacity and cytotherapeutic potential of periodontal ligament cells for periodontal regeneration in vitro and in vivo

**DOI:** 10.7717/peerj.6589

**Published:** 2019-03-08

**Authors:** Jinghui Li, Fangming Zhang, Ning Zhang, Xuefei Geng, Cen Meng, Xiaoying Wang, Ying Yang

**Affiliations:** Department of stomatology, Beijing Friendship Hospital, Capital Medical University, Beijing, China

**Keywords:** Periodontal regeneration, Human periodontal ligament cells (PDLCs), Human bone marrow derived mesenchymal stem cells (hMSCs), Osteogenic differentiation

## Abstract

**Background:**

The periodontal ligament cells (PDLCs) contain heterogeneous cell populations and possess stem-cell-like properties. PDLCs have attracted considerable attention as an option for periodontal regeneration. However, the osteogenic differentiation of PDLCs remains obscure owing to variable osteo-inductive methods and whether PDLCs could be directly used for periodontal regeneration without stem cell enrichment is uncertain. The aim of the present study was to clarify the osteogenic differentiation capacity of PDLCs and test PDLCs as an alternative to stem cells for periodontal regeneration.

**Methods:**

We tested the performance of human PDLCs in osteo-inductive culture and transplantation in vivo while taking human bone marrow derived mesenchymal stem cells (hMSCs) as positive control. Proliferation of PDLCs and hMSCs in osteo-inductive condition were examined by MTT assay and colony formation assay. The osteogenic differentiations of PDLCs and hMSCs were assessed by Alkaline phosphatase (ALP) activity measurement, von Kossa staining, Alizarin red S staining and quantitative RT-PCR of osteogenic marker gene including RUNX2, ALP, OCN, Col I, BSP, OPN. We transplanted osteo-inductive PDLCs and hMSCs with hydroxyapatite/tricalcium phosphate (HA/TCP) scaffolds to immunodeficient mice to explore their biological behaviors in vivo by histological staining and immunohistochemical evaluation.

**Results:**

After 14 days of osteo-induction, PDLCs exhibited significantly higher proliferation rate but lower colony-forming ability comparing with hMSCs. PDLCs demonstrated lower ALP activity and generated fewer mineralized nodules than hMSCs. PDLCs showed overall up-regulated expression of RUNX2, ALP, OCN, Col I, BSP, OPN after osteo-induction. Col I level of PDLCs in osteo-inductive group was significantly higher while RUNX2, ALP, OCN were lower than that of hMSCs. Massive fiber bundles were produced linking or circling the scaffold while the bone-like structures were limited in the PDLCs-loaded HA/TCP samples. The fiber bundles displayed strong positive Col I, but weak OCN and OPN staining. The in vivo results were consistent with the in vitro data, which confirmed strong collagen forming ability and considerable osteogenic potential of PDLCs.

**Conclusion:**

It is encouraging to find that PDLCs exhibit higher proliferation, stronger collagen fiber formation capacity, but lower osteogenic differentiation ability in comparison with hMSCs. This characteristic is essential for the successful periodontal reconstruction which is based on the synchronization of fiber formation and bone deposition. Moreover, PDLCs have advantages such as good accessibility, abundant source, vigorous proliferation and evident osteogenic differentiation capacity when triggered properly. They can independently form PDL-like structure in vivo without specific stem cell enrichment procedure. The application of PDLCs may offer a novel cytotherapeutic option for future clinical periodontal reconstruction.

## Introduction

Periodontal diseases are common and are the main reasons for teeth loss worldwide. The progression of periodontal disease results in destruction of tooth supporting tissues involving alveolar bone, periodontal ligament (PDL) and cementum. Retaining or improving PDL function is crucial for restoring periodontal defects. Many periodontal tissue engineering approaches have been proposed such as guided tissue regeneration (GTR), growth factor utilization like EMD, BMPs, FGF and cell transplantation, etc. (Andrei et al., 2018; Siaili, Chatzopoulou & Gillam, 2018). GTR and growth factor utilization are currently applied clinically but only result in partial regeneration which may be due to the unique periodontal anatomical structure and different alveolar bone defect morphology (Ripamonti & Petit, 2009; Bottino et al., 2012; Aimetti et al., 2016; Soltani Dehnavi et al., 2018). Therefore, suitable seed cells and efficient cytotherapeutic approaches are imperative for the regeneration of periodontium.

Several mesenchymal-stem-like cell populations have been successfully derived from different dental tissues such as dental pulp stem cells, stem cells from exfoliated deciduous teeth, PDL stem cells, stem cells from apical papilla and dental follicle progenitor cells (Rodríguez-Lozano et al., 2011). These stem cells, as well as those from bone marrow (Hasegawa et al., 2006; Yu et al., 2013) and adipose tissue (Sugawara & Sato, 2014; Suzuki et al., 2017) are good therapeutic candidates for periodontal regeneration. But their application is greatly limited by the source shortage, low harvesting efficiency and difficulty in isolation, culture and proliferation.

The PDLCs consisted of heterogeneous cell populations including fibroblasts, endothelial cells, epithelial cell rests of Malassez, sensory cells (such as Rufini-type end organ receptors), osteoblasts and cementoblasts (D’Errico et al., 1999; Lekic et al., 2001; Xiong, Gronthos & Bartold, 2013). PDLCs possess distinct functional characteristics and differ from cells in other connective tissues in a number of respects. Many studies showed that PDLCs had stem cell properties and performed osteogenic differentiation when triggered appropriately (Seo et al., 2004; Nagatomo et al., 2006; Silvério et al., 2010; Moshaverinia et al., 2014a). PDLCs have attracted considerable attention as an alternative to mesenchymal stem cells owing to their promising osteogenic differentiation ability, high proliferation rate, easy accessibility and abundant quantity (Akizuki et al., 2005; Iwata et al., 2010; Zhou et al., 2012). However, the osteogenic differentiation of PDLCs remains obscure owing to variable osteo-inductive methods and whether PDLCs could be directly used for periodontal regeneration without the enrichment of mesenchymal-stem-like cells is uncertain (Yang et al., 2013; Seubbuk et al., 2017; Wei et al., 2017; Proksch et al., 2018).

The aim of the present study was to clarify the osteogenic differentiation capacity of PDLCs comparing with human bone marrow derived mesenchymal stem cells (hMSCs) under easy-handling, practical and standardized osteo-inductive procedures in vitro and in vivo. We tested PDLCs as an alternative source for periodontal tissue regeneration by transplanting PDLCs-loaded hydroxyapatite/tricalcium phosphate (HA/TCP) scaffold into immunodeficient mice. We anticipated that this study may provide us with a deeper understanding of the biological properties and application potential of PDLCs for future periodontal tissue engineering and regeneration.

## Materials and Methods

### Cell culture and osteogenic differentiation

Human PDLCs were isolated from healthy permanent premolars extracted for orthodontic reasons (10–14-year-old donors) in Department of stomatology, Beijing Friendship Hospital, Capital Medical University. Following surgical extraction, soft tissues close to root neck or apical zone were removed. Extracted tooth roots with the middle part of PDL were digested with an enzyme mixture (two mg/mL dispase, one mg/mL collagenase type I) at 37 °C for 1.5 h. PDLCs pellets were then collected by centrifugation at 1,000 rpm for 10 min. The pellets were re-suspended with dulbecco’s modified eagle media (DMEM) containing 10% fetal bovine serum and 100 U/mL penicillin and 100 μg/mL streptomycin (Invitrogen, Carlsbad, CA, USA), and cultured in a humidified atmosphere of 5% CO_2_ at 37 °C. The study was approved by the Ethics Review Board of Beijing Friendship Hospital, Capital Medical University, Beijing, China (2017-P2-109-01).

Human bone marrow derived mesenchymal stem cells were obtained from inpatients of Beijing Friendship Hospital, Capital Medical University who suffered alveolar cleft (11–20-year-old donors). Approximately 10 mL bone marrow of iliac crest was obtained and kept in 10 mL Hanks solution with 100 U/mL sodium heparin. The hMSCs-enriched low-density fraction was collected by centrifuging in lymphocyte separation medium at 1,000 rpm for 10 min. The hMSCs were re-suspended with DMEM containing 10% fetal calf serum, 100 U/mL penicillin and 100 μg/mL streptomycin (Invitrogen, Carlsbad, CA, USA).

The four to six passages of PDLCs and hMSCs were used for the following experiments. Osteogenic differentiation was performed by changing the culture medium to differentiation medium, comprising of DMEM supplemented with 100 nM Dexamethasone, 10 mM sodium β-glycerophosphate and 50 nM ascorbic acid-2-phosphate (Thermo Fisher, Waltham, MA, USA). Differentiation medium was replenished every 3 days during a differentiation period of 14 days.

### MTT assay

Periodontal ligament cells and hMSCs were seeded in 96-well plates at a density of 1 × 10^3^ cells/well and induced to osteogenic differentiation. Growth profiles of the cells were determined using MTT method, during a course of 8 days. At each time point, 20 μL of MTT solution (3-(4,5-dimethylthiazol-2-yl)-2,5-diphenyltetrazolium bromide) was added to the cells in each well, incubated at 37 °C for 4 h, replaced the medium with 150 μL dimethyl sulfoxide (DMSO) (Sigma, St. Louis, MO, USA) at the end of incubation. Analysis were performed with a microplate reader (ELX800; BioTek Instruments Inc, Winooski, VT, USA) at 570 nm, to calculate the profiles of cell proliferation.

### Colony formation assay

To assess the colony-forming capability of PDLCs and hMSCs, single cell suspensions were seeded into Φ6 cm dishes (Costar, Washington, D.C., USA) at a density of 500 cells/dish. After induced osteogenic differentiation of 14-day, the dishes were washed with PBS for two times, fixed in ice-cold methanol for 10 min, and stained with crystal violet (Sigma, St. Louis, MO, USA). Photographs were taken with a phase-contrast microscope (Nikon, Tokyo, Japan) and quantitative analysis of colony formation was carried out using ImageJ (Rasband, 2016).

### Alkaline phosphatase staining

Periodontal ligament cells and hMSCs were seeded in 12-well plates (Costar, Washington, D.C., USA) at a density of 5 × 10^4^ cells/well. After osteogenic differentiation performed as described, Alkaline phosphatase (ALP) staining was carried out. In brief, culture wells were rinsed twice with PBS, fixed in 95% alcohol for 1 min, washed twice with distilled water, incubated with substrates mixture (2% sodium β-glycerophosphate 25 mL, 2% sodium barbiturate 25 mL, distilled water, 50 mL, 2% calcium chloride five mL, 2% magnesium sulfate two mL, several drops of chlorform) at 37 °C for 3 h, washed twice with distilled water, incubated in 2% cobalt nitrate for 2 min, and finally incubated with 2% amine sulfide for 1 min. Staining pictures were taken using a phase-contrast microscope (Nikon, Tokyo, Japan).

### Measurement of ALP activity

Periodontal ligament cells and hMSCs were seeded in 24-well plates (Costar, Washington, D.C., USA) at 1 × 10^4^ cells/well. Osteogenic differentiation was performed as described. After a culture of 14 days, cells were collected and lysed in 0.2% Triton X-100 lysis buffer. With cell lysates, ALP activity was measured using a StemTAG™ Alkaline Phosphatase Activity Assay Kit (CBA-301; Cell biolabs, San Diego, CA, USA). Protein concentration was determined using a Pierce BCA Protein Assay Kit (Thermo Fisher, Waltham, MA, USA), and was used to normalize ALP activity for each sample.

### Mineralization assay—Von kossa staining and Alizarin red S staining

The PDLCs and hMSCs were seeded at 5 × 10^4^ cells/well in 12-well plates (Costar, Washington, D.C., USA) respectively. After 14 days of osteo-incubation, Von kossa staining was performed by means of a modified method (Cheng et al., 1994). In brief, culture wells were rinsed with PBS, fixed with 95% alcohol for 1 min, rinsed three times with deionized water, incubated in flesh 2% silver nitrate at 37 °C for 30 min, washed three times with deionized water, and exposed to UV-light for 30 min to present the results.

For Alizarin Red S Staining, cells were fixed with cold methanol at room temperature for 10 min, rinsed twice with distilled deionized water, stained with 1% Alizarin Red S (pH4, Sigma, St. Louis, MO, USA) at room temperature for 30 min. Then, the excess dye was removed and the cells were rinsed with distilled deionized water until the background became clear.

### Osteogenic marker genes expression—Quantitative RT-PCR assay

To analyze the osteogenic marker genes expression in PDLCs and hMSCs following the induction of osteogenic medium for 14 days, total RNAs were isolated using TRIzol reagent (Invitrogen, Carlsbad, CA, USA). cDNA was synthesized using Script M-MLV reagent (Invitrogen, Carlsbad, CA, USA). Briefly, 15 μL reverse transcription reactions containing one μg total RNA, two μL oligo-dT primer, two μL deoxynucleotide (dNTP) (10 mM each), four μL 5 × first-strand buffer, 0.5 μL RNase inhibitor (40 U/μL), and one μL TIANScript M-MLV (200 U/μL). For reverse transcription, the mixture was incubated at 70 °C for 5 min and moved onto ice for 2 min and then incubated at 25 °C for 10 min, 42 °C for 50 min, and finally 95 °C for 5 min. Osteogenic markers of RUNX2 (runt-related transcription factor 2), ALP, OCN (osteocalcin), COL1 (collagen type I), BSP (bone sialoprotein), OPN (osteopontin) were examined by quantitative real-time polymerase chain (PCR) reaction. β-actin was used as an internal reference in all applications. Specific primers were synthesized by Sangon Biotech, China (Table 1). The real-time PCR (TIANGEN, Beijing, China) was performed using a 25 μL PCR reaction with two μL RT product, 2.5 μL 10 × HotMaster Taq buffer, one μL dNTP (2.5 mM each), and 0.2 μL HotMaster Taq polymerase (2.5 U/μL). The reactions were carried out at 95 °C for 2 min, followed by 40 cycles of 95 °C for 30 s, 60 °C for 30 s, and 72 °C for 30 s. Relative gene expression was calculated by the 2^−ΔΔCT^ method.

**Table 1:** Specific primers used for real-time polymerase chain reaction of osteogenic markers.

Primer	Abbreviation	GenBank accession	Amplicon Size (bp)	Sequence (5′ ≥ 3′)
Runt-related transcription factor 2	RUNX2	NM_001015051	101	Forward: TGGTTACTGTCATGGCGGGTA
				Reverse: TCTCAGATCGTTGAACCTTGCTA
Alkaline phosphatase	ALP	NM_001632	81	Forward: GTGAACCGCAACTGGTACTC
				Reverse: GAGCTGCGTAGCGATGTCC
Osteocalcin	OCN	NM_199173	112	Forward: CACTCCTCGCCCTATTGGC
				Reverse: CCCTCCTGCTTGGACACAAAG
Collagen type I	Col I	NM_000088	140	Forward: GAGGGCCAAGACGAAGACATC
				Reverse: CAGATCACGTCATCGCACAAC
Bone sialoprotein	BSP	NM_004967	106	Forward: CACTGGAGCCAATGCAGAAGA
				Reverse: TGGTGGGGTTGTAGGTTCAAA
Osteopontin	OPN	NM_001251830	230	Forward: CTCCATTGACTCGAACGACTC
				Reverse: CAGGTCTGCGAAACTTCTTAGAT
Beta actin	β-actin	NM_001101	250	Forward: CATGTACGTTGCTATCCAGGC
				Reverse: CTCCTTAATGTCACGCACGAT

### Transplantation

#### Preparation for transplantation vehicles

Hydroxyapatite/tricalcium phosphate ceramics scaffolds were incubated in serum-free DMEM medium for 2 days and then sterilized under 121 °C for 15 min. Osteogenic differentiation was performed with PDLCs and hMSCs for a period of 14 days, 2 × 10^5^ cells were then loaded to a piece of HA/TCP of 30 mm^3^ volume and transplanted to immunodeficient mice respectively.

#### Surgical implantation procedures

Immunodeficient male severe combined immunodeficient mice (SCID) mice of 5 week-old (*n* = 6) were used for transplant recipients of PDLCs or hMSCs. Operation was performed under local anesthesia made by lidocaine hydrochloride injection. Skin incision was made on the dorsal surface of each mouse, subcutaneous pockets were made in which cell-loaded HA/TCP scaffolds were placed. Up to four transplants were inoculated per animal. The experimental protocol was approved by the Institutional Animal Care and Use Committee of Beijing Friendship Hospital, Capital Medical University, Beijing, China (18-2004).

#### Histological and immunohistochemical assay

Mice were sacrificed 12 weeks after transplantation. Tissue samples were surgical isolated, fixed in 4% formalin for 24 h, decalcified in 14% EDTA solution (pH 7.0) for 7–10 days, and embedded in paraffin. After tissue sectioning (four μm in thickness), slides were deparaffinized, hydrated and stained with hematoxylin and eosin (HE) using standard techniques. For immunohistochemical staining, slides were heated in a 60 °C oven for 30 min, and subsequently hydrated to water through a series of decreasing concentrations of ethanol. Then immunohistochemical staining was performed using a Immunohistochemical kit SP-9001 (Zhongshan Biotech Co., Zhongshan, China). The used anti-OCN, anti-COL1, anti-OPN antibodies were purchased from Cell Signaling Technology (CST, Danvers, MA, USA). Results were observed and documented using an Olympus BX60 microscope.

### Statistical analysis of data

Statistic analyses were performed with Student’s *t*-test, *n* ≥ 3. Data are presented as mean ± standard deviation. Differences between groups are considered statistically significant if *P* < 0.05.

## Results

### Morphology and proliferation of PDLCs and hMSCs in osteo-inductive condition

**Figure 1:**
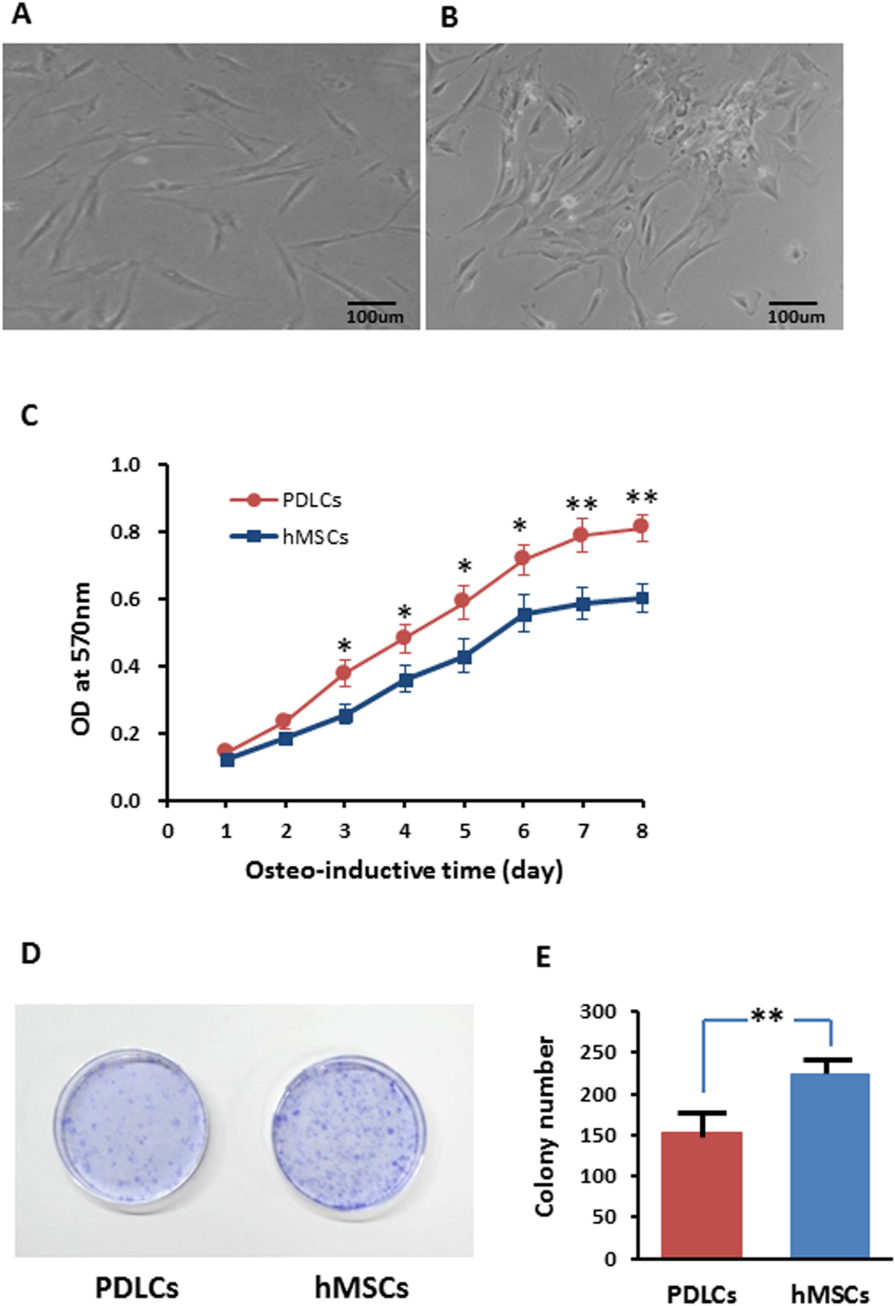
Morphology and proliferation of PDLCs and hMSCs in osteo-inductive condition. (A) PDLCs’ morphology after 14 days of osteo-inductive culture. (B) hMSCs’ morphology after 14 days of osteo-inductive culture. (C) Proliferation curve of PDLCs and hMSCs under osteo-inductive culture. (D) Colony formation assay of PDLCs and hMSCs after 14 days of osteo-inductive culture. (E) Quantitative analysis of colony formation. Scale bars, 100 μm. (Student’s *t*-test, *n* ≥ 3; *P < 0.05, **P < 0.01).

Periodontal ligament cells presented fibroblast-like morphology while after 14 days of osteo-inductive culture (Fig. 1A) while about half percentage of hMSCs exhibited relatively flattened broad shape (Fig. 1B). PDLCs demonstrated significantly higher proliferation rate but lower colony-forming ability comparing with hMSCs in osteo-inductive condition (Figs. 1C–1E; *P* < 0.01). The higher self-renewal efficiency of PDLCs provided a potential superiority in periodontal tissue regeneration.

### Osteogenic differentiation in vitro

Periodontal ligament cells and hMSCs were exposed to osteogenic medium for 14 days and then osteogenic differentiations were assessed by measuring ALP activity, calcium deposition and osteogenic marker gene expression.

#### Alkaline phosphatase activity

**Figure 2:**
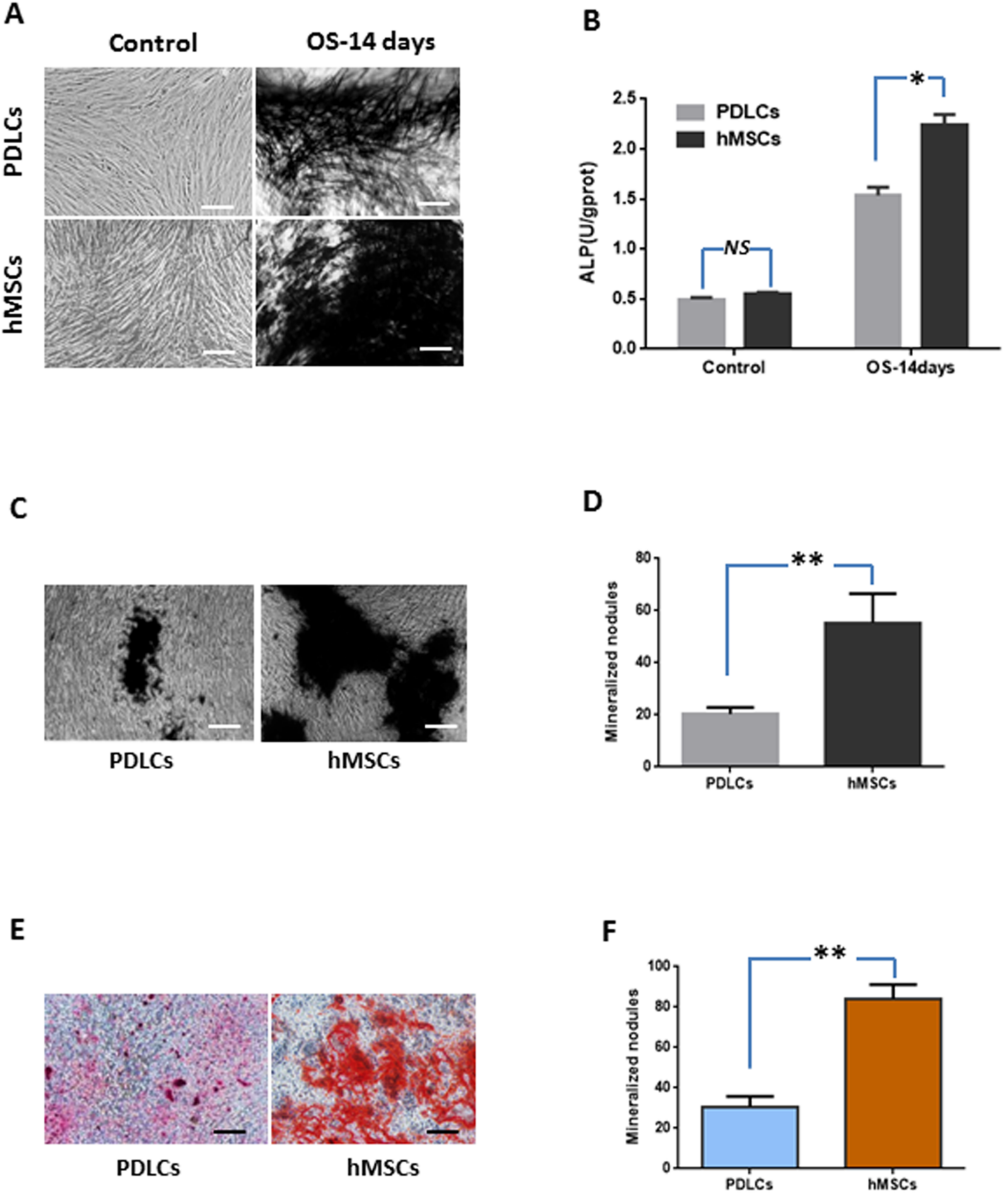
Osteogenic differentiation of PDLCs and hMSCs after 14 days of osteo-inductive incubation in vitro. (A) ALP staining. The PDLCs and hMSCs kept in normal growth medium were used as control, while the PDLCs and hMSCs kept in osteo-inductive medium were assessed as test groups. (B) ALP activity assay. (C) von Kossa staining of PDLCs and hMSCs after 14 days of osteo-inductive incubation. (D) Quantitative analysis of von Kossa staining. (E) Alizarin red S staining of PDLCs and hMSCs after 14 days of osteo-inductive incubation. (F) Quantitative analysis of Alizarin red S staining. Scale bars, 100 μm. (Student’s *t*-test, *n* ≥ 3; *P < 0.05, **P < 0.01).

Under normal culture condition, both PDLCs and hMSCs showed very weak ALP staining and low ALP activity. While in osteo-inductive medium, PDLCs and hMSCs both demonstrated strong ALP-positive staining and revealed higher ALP activity compared to their control, respectively (*P* < 0.05). No significant difference was found between PDLCs and hMSCs control group (*P* = 0.45), but the ALP activity of hMSCs was higher than that of PDLCs in osteo-inductive groups (Figs. 2A and 2B; *P* < 0.05).

#### Calcium deposition

Calcium deposition were further assessed by von Kossa staining and Alizarin red S staining after 14 days of osteogenic culture. Osteo-induction produced mineralized nodules in both PDLCs and hMSCs (Figs. 2C–2F). PDLCs yielded smaller and fewer mineralized nodules comparing with hMSCs under osteo-inductive condition. Quantitative analysis also convinced the relatively inferior calcified capacity of PDLCs compared to hMSCs (*P* < 0.01).

#### Osteogenic marker genes expression

**Figure 3:**
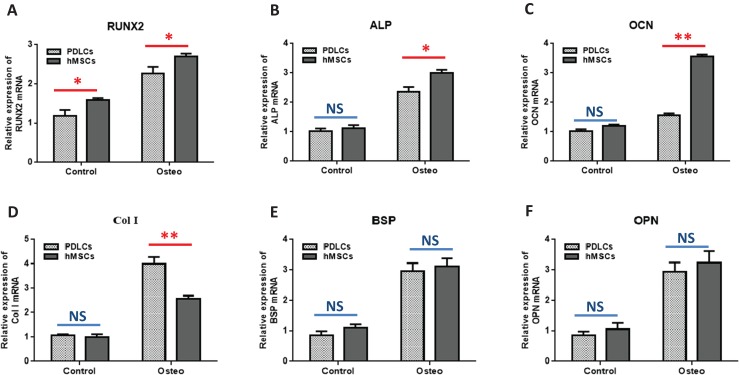
Osteogenic marker genes expression in PDLCs and hMSCs after 14 days of osteo-inductive incubation in vitro. The PDLCs and hMSCs kept in normal growth medium were used as control, while the PDLCs and hMSCs kept in osteo-inductive medium were assessed as test groups. The expression of RUNX2(A), ALP(B), OCN(C), Col I(D), BSP(E), OPN(F) were analyzed by quantitative real-time PCR. (Student’s *t*-test, *n* ≥ 3; *P < 0.05, **P < 0.01).

Quantitative RT-PCR showed that the expression levels of osteoblast-specific genes, such as RUNX2, ALP, OCN, Col I, BSP, OPN, were generally higher in both PDLCs and hMSCs after 14 days of osteo-inductive medium incubation (Osteo group) than those in normal medium (Control group) (Fig. 3; *P* < 0.05). No significant differences were found in control groups of PDLCs and hMSCs when they were cultured in normal medium (*P* > 0.05) except hMSCs exhibited a slightly increased expression of RUNX2 (Fig. 3A; *P* < 0.05). When cultured in Osteo-inductive medium, PDLCs showed a promising osteogenic ability by the overall up-regulated expression of RUNX2, ALP, OCN, Col I, BSP, OPN (*P* < 0.05). Comparing with hMSCs counterparts, PDLCs (Osteo group) demonstrated a little bit lower expression of RUNX2, ALP, OCN (Fig. 3A–3C; *P* < 0.05) but almost the same level of BSP, OPN (Figs. 3E and 3F; *P* > 0.05). This confirmed that PDLCs may differentiate toward osteoblastic direction under proper inductive condition. Furthermore, Col I level of PDLCs (Osteo group) was significantly higher than that of hMSCs which indicated PDLCs possessed better collagen forming capacity (Fig. 3D; *P* < 0.01).

### Osteogenic differentiation in vivo

**Figure 4:**
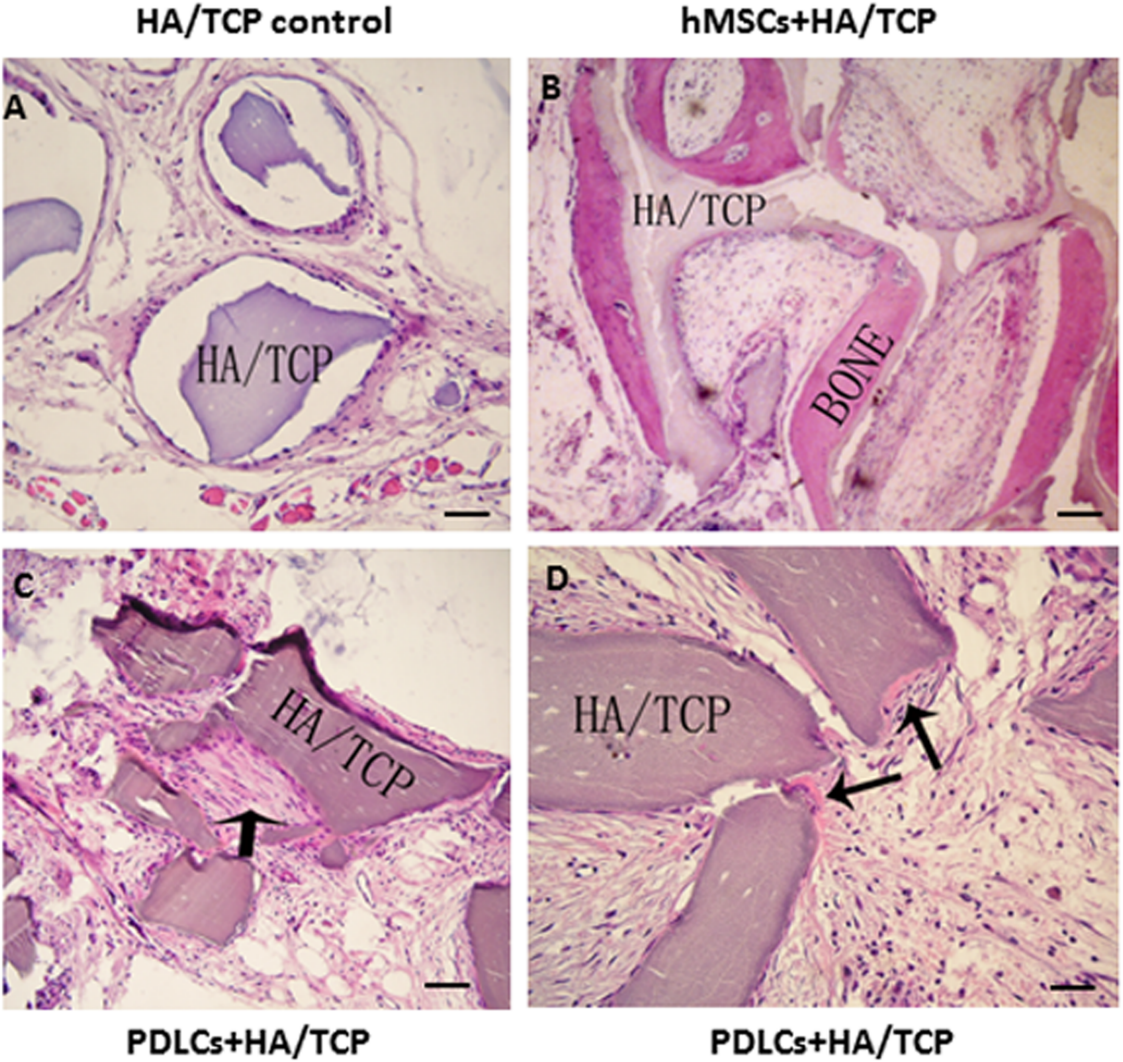
Transplantation of osteo-inductive PDLCs and hMSCs with HA/TCP scaffolds to SCID mice. After 12 weeks, the transplants were taken out and performed HE staining. (A) Cell-free HA/TCP control. No evident bone formed or fiber cluster appeared. (B) In hMSCs-loaded HA/TCP samples, ectopic bone formation was found, but no obvious fiber bundles appeared. (C–D) In the PDLCs-loaded HA/TCP samples, massive fiber bundles linked or circled the scaffold (C, arrow) were found while the bone-like structure was limited (D, arrows). Scale bars, 100 μm.

**Figure 5:**
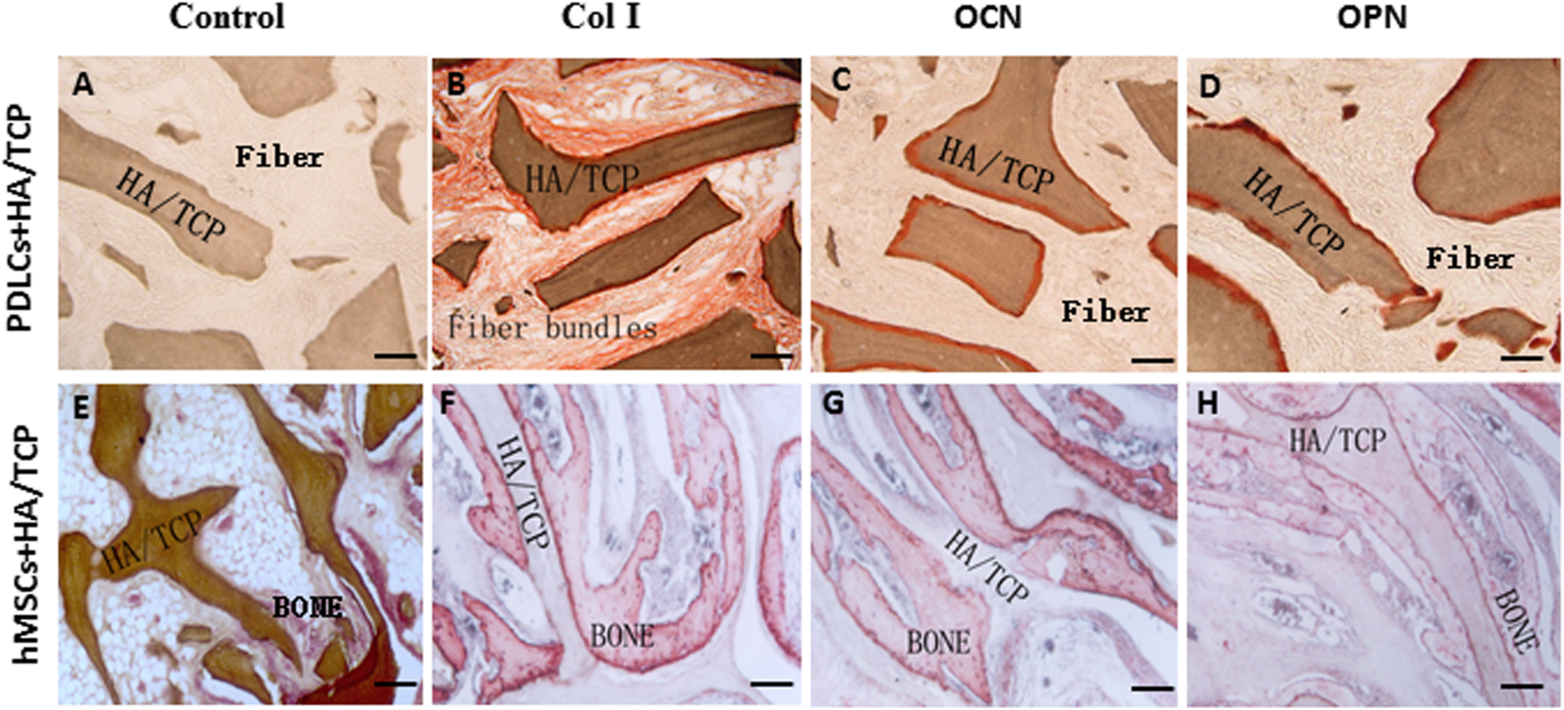
Osteogenic differentiation of PDLCs and hMSCs in vivo. The expression of Col I, OCN and OPN in the transplants were evaluated by immunohistochemical staining. (A–D) In the PDLCs-loaded HA/TCP samples, the fiber bundles displayed strong positive Col I(B), but weak OCN(C) and OPN(D) staining. (E–H) In the hMSCs-loaded HA/TCP samples, the ectopic bone structure exhibited positive staining of Col I(F), OCN(G) and OPN(H). This result confirmed strong collagen forming ability and considerable osteogenic potential of PDLCs in vivo. Scale bars, 100 μm.

To explore the application possibility of PDLCs and hMSCs in periodontal regeneration, we triggered the osteogenic differentiation of PDLCs and hMSCs by culturing them in osteo-inductive medium for 14 days in vitro. And then we transplanted the osteo-inductive cells with HA/TCP scaffolds to SCID mice is to examine their biological behaviors in vivo. A total of 12 weeks later, the transplants were taken out and put through histological evaluation by HE staining (Fig. 4) and Col I, OCN and OPN immunohistochemical staining (Fig. 5). There was no evident bone formed or fiber cluster appeared in cell-free HA/TCP control (Fig. 4A). Ectopic bone formation was found in the 12-week hMSCs-loaded HA/TCP, while no fiber bundles were formed (Fig. 4B). On the contrary, massive fiber bundles were produced linking or circling the scaffold while the bone-like structures were limited in the PDLCs-loaded HA/TCP samples (Figs. 4C and 4D). The fiber bundles displayed strong positive Col I, but weak OCN and OPN staining (Figs. 5B–5D). The ectopic bone structure in hMSCs-loaded HA/TCP samples exhibited positive staining of Col I, OCN and OPN (Figs. 5F–5H). The in vivo results were consistent with the in vitro data which confirmed strong collagen forming ability and considerable osteogenic potential of PDLCs.

## Discussion

Periodontal diseases are mainly inflammatory diseases and eventually lead to the destruction of periodontal tissues and teeth loss. Aside from the initial periodontal therapy such as scaling, root planning and periodontal surgical treatment to remove the infectious tissue, the ultimate therapeutic goal is to restore the lost fibrous PDL attachment and form new bone and cementum (Estrela et al., 2011; Han et al., 2014a). For this purpose, several periodontal tissue engineering approaches have been proposed, including genetic engineering, growth factor utilization, and cell transplantation. An ideal cell transplantation approach requires adequate source cells which are accepted by the recipient immune system (Maeda et al., 2011; Fu et al., 2014; Liu et al., 2015). Considering that periodontal tissue consists of gingiva, alveolar bone, cementum and PDL, it is better to choose periodontal-derived cells and the heterogeneous resource cell population may be favorable for periodontal regeneration (Akizuki et al., 2005; Iwata et al., 2010). To repair periodontal defects, the regeneration of the PDL is as important as reparation of the bone. Focus should be put on both bone regeneration and functionally oriented PDL. To this aspect, PDLCs may provide an ideal resource cells due to their abundance and heterogeneous components (Huang, Gronthos & Shi, 2009; Hynes et al., 2012; Iwasaki et al., 2014). But the effectiveness is unclear and the successful rate is unpredictable. Our objective was trying to illuminate the regenerating capacity of PDLCs and optimize the procedures.

The PDLCs are heterogeneous mixture in which the predominant cell type is fibroblasts (McCulloch & Bordin, 1991). The PDL fibroblasts originate from the ectomesenchyme of the investing layer of the dental papilla and dental follicle. Whether these fibroblast subsets are derived from one single type of progenitor cell is unknown but they exhibit different functional characteristics (McCulloch, 1995; Lekic & McCulloch, 1996). We found that PDLCs exhibited considerably higher proliferation rate but lower colony-forming ability comparing with hMSCs in osteo-inductive condition. This may be partially due to the major percentage of fibroblasts in PDLCs mixture. The higher self-renewal efficiency of PDLCs provided a potential superiority in periodontal tissue regeneration. Moreover, PDLCs are relatively abundant in extracted teeth and can be non-invasively obtained comparing with hMSCs.

However, the regenerative properties of PDLCs are affected by donor age, disease condition and tissue quality. PDLCs derived from young donors revealed greater cementum- and PDL-like tissue formation than those from aged donors. The self-renewal capacity of PDLCs decreased while donor aging (Gao et al., 2013; Tomokiyo et al., 2018). Inflammation is another factor to affect the regenerative potential of PDLCs. PDLCs obtained from the patients with periodontitis showed significantly lower bone formation than the cells acquired from the patients with healthy PDL tissues (Tang et al., 2016). Therefore, how to obtain large numbers of PDLCs and maintain good self-renewal capacity is significantly important to achieve PDL regenerative therapy especially in elderly patients since prevalence and severity of periodontal disease are increasing with age. More efforts should be devoted to this issue for future clinical applications.

In addition, different cell harvesting methods lead to different cell components which eventually result in different tissue engineering outcomes (Seo et al., 2004; Proksch et al., 2018). We obtained the PDLCs from the extracted roots digested for 1.5 h at 37 °C with enzyme mixture. Under this condition, we might harvest more fibroblasts from the PDL than cementoblasts which attach to the root surface or osteoblasts which anchor to the alveolar bone. After transplantation, these isolated PDLCs got a typical PDL-like structure in vivo.

Because of the heterogeneity of PDLCs, there is contradictory evidence about whether PDLCs have the capacity to differentiate into osteoblast or form hard tissue following osteogenic induction. Many researchers reported that PDLCs had the ability to differentiate into osteoblast-like cells when triggered appropriately (Fuchigami et al., 2016; Seubbuk et al., 2017; Furue et al., 2017). However, the osteo-inductive methods were highly variable and certain experimental perturbations existed which might impair the validity of the results (Hu et al., 2014; An et al., 2015; Seubbuk et al., 2017; Wei et al., 2017). Therefore, in this study, we uniformed the osteo-inductive condition for both PDLCs and hMSCs in order to make the results more clear and definite. We found that PDLCs expressed significantly elevated ALP activity, calcium deposition and osteogenic marker gene expression (RUNX2, ALP, OCN, Col I, BSP, OPN) after 14 days of osteo-induction. This confirmed that PDLCs actually had the osteogenic differentiation ability under proper inductive condition, although which was a little bit inferior to hMSCs since the mineralized nodules in vitro and bone-like structure in vivo of PDLCs samples were fewer than that of hMSCs.

A wide variety of animal models (dog, rat, pig and sheep) and defect types were created to explore the successful periodontal regeneration with use of PDLCs periodontal ligament stem cells and scaffolds (Han et al., 2014b; Bright et al., 2015; Tassi et al., 2017). Owing to the wide variability in defect type, cell source and cell scaffold, no meta-analysis or evident conclusion was possible (Bright et al., 2015). Due to the different surgical procedures to establish animal models for periodontal defects, it was also hard to tell whether the bone/cementum formation owes to the transplanted PDLCs differentiation or just repairation from the residue PDL structure. We performed xenotransplantation of human PDLCs-loaded scaffold to immunodeficient mice subcutaneously to clarify the pure self-rebuild function of PDLCs and excluded the interference from adjacent tissue cells. Evident bone formation was found in the 12-week hMSCs-loaded HA/TCP transplants, while no fiber bundles were formed. In contrast, massive fiber bundles were produced linking or circling the scaffold while the bone-like structure was limited in the PDLCs-loaded HA/TCP samples. The fiber bundles displayed strong positive Col I and weak OCN, OPN staining. Our in vitro and in vivo data showed that PDLCs demonstrated superior collagen forming ability comparing to hMSCs. The abundant collagen type I-positive oriented fiber bundles formed by PDLCs were preferable for the future reconstruction of PDL structure. This was consistent with the studies conducted by other scholars in which the favorable fiber-forming ability of PDLCs has already been successfully applied in the repair of tendon injuries (Moshaverinia et al., 2014b; Hsieh et al., 2016).

Besides the inherent variant properties of PDLCs and hMSCs, another reason accounting for the different differentiation capacity may be the local regeneration niche (Dangaria et al., 2009). We even transplanted PDLCs into the periodontal defect animal models, we could not simulate the micro-environment exactly the same as that of the human periodontium. The scaffold architecture should be optimized to fulfill proper spacious distribution and physiological force loading function. This requires further and intensive research.

In summary, the successful reconstruction of periodontium is based on the synchronization of fiber forming and bone depositing which enables the anchorage of PDL into cementum and alveolar bone. Abundant source, good proliferation, combination of powerful fiber forming capacity and considerable osteogenic differentiation ability provide PDLCs as a novel candidate for periodontal regeneration.

## Conclusions

Compared with hMSCs, PDLCs are easier to acquire and provide an abundant source for cell-based periodontal regeneration. PDLCs exhibit vigorous proliferation, evident osteogenic differentiation capacity and strong fiber-forming ability when triggered properly. They can independently form PDL-like structures in vivo without specific stem cell enrichment procedure. The application of PDLCs may offer a novel cytotherapeutic option for future clinical periodontal reconstruction.

## Supplemental Information

10.7717/peerj.6589/supp-1 Supplemental Information 1 Raw data.

## Additional Information and Declarations

### Data Availability

The following information was supplied regarding data availability: The raw data are available in the Supplemental File.
